# Refractory Dysphagia and Severe Iron Deficiency Anemia in a Woman With Plummer-Vinson Syndrome

**DOI:** 10.7759/cureus.116157

**Published:** 2026-09-12

**Authors:** Numan Kavaz

**Affiliations:** 1 Internal Medicine, Gaziosmanpaşa Training and Research Hospital, University of Health Sciences, Istanbul, TUR

**Keywords:** dysphagia, endoscopic dilatation, esophageal web, iron deficiency anemia (ida), plummer-vinson syndrome

## Abstract

Plummer-Vinson syndrome (PVS) is a rare clinical entity defined by the triad of iron deficiency anemia, dysphagia, and an upper esophageal web. Its prevalence has declined worldwide in parallel with improvements in nutrition, and the condition is therefore encountered infrequently in contemporary practice. Because PVS carries an increased risk of squamous cell carcinoma of the hypopharynx and upper esophagus, timely recognition and long-term surveillance are important. We report a 47-year-old woman who presented with a four-month history of progressive dysphagia that had not responded to a one-month course of proton pump inhibitor (PPI) therapy prescribed at another center. Laboratory evaluation revealed severe microcytic hypochromic anemia with a hemoglobin level of 7 g/dL (reference range: 12.0-16.0 g/dL), a mean corpuscular volume (MCV) of 52 fL (reference range: 80-100 fL), and a ferritin level of 3 ng/mL (reference range: 15-150 ng/mL). Intravenous ferric carboxymaltose was administered, and upper gastrointestinal endoscopy demonstrated a web at the esophageal inlet, which was dilated during the same session. By the third month, the hemoglobin level had risen to 13 g/dL, with complete resolution of dysphagia. This case illustrates that progressive dysphagia unresponsive to antisecretory therapy is an alarm symptom warranting prompt endoscopic evaluation, and that a simple complete blood count may provide the decisive diagnostic clue in such patients.

## Introduction

Plummer-Vinson syndrome (PVS), also referred to as Paterson-Brown Kelly syndrome, is characterized by the triad of dysphagia, iron deficiency anemia, and an esophageal web in the post-cricoid region [1,2]. Although described more than a century ago, the condition is now considered rare, and its prevalence appears to have declined worldwide, most likely as a consequence of improved nutritional status and the wider availability of iron supplementation [2,3].

The etiopathogenesis of PVS remains incompletely understood. Iron deficiency is regarded as the most important contributing factor, while malnutrition, genetic predisposition, and autoimmune mechanisms have also been proposed [1]. The prevailing hypothesis holds that depletion of iron-dependent oxidative enzymes leads to atrophy of the esophageal mucosa and subsequent web formation in a mucosal surface characterized by high cell turnover [4]. Consistent with a functional contribution of iron deficiency, esophageal motility abnormalities in these patients have been shown to improve following iron replacement [5].

Most patients are middle-aged women, and the dysphagia is typically painless, limited to solids, and either intermittent or slowly progressive [1]. Symptoms attributable to anemia may dominate the clinical picture and divert attention from the esophageal component. Importantly, PVS has been associated with an increased risk of squamous cell carcinoma of the pharynx and esophagus, and long-term clinical follow-up with consideration of endoscopic surveillance is therefore advised, although optimal surveillance intervals are not well established [1,6]. Treatment rests on iron replacement, with mechanical dilatation reserved for patients in whom the web produces significant luminal obstruction or in whom dysphagia persists despite iron repletion [1,7].

Herein, we present a patient referred with progressive dysphagia unresponsive to empirical antisecretory therapy, in whom PVS was diagnosed and managed successfully with a combination of intravenous iron replacement and endoscopic dilatation.

## Case presentation

A 47-year-old woman presented to our outpatient clinic with a four-month history of progressive difficulty in swallowing. The symptom had initially been intermittent and confined to solid food but had become steadily more troublesome and was accompanied by odynophagia. There was no weight loss and no symptom suggestive of aspiration. She had been evaluated at another center, where a one-month course of proton pump inhibitor (PPI) therapy had been prescribed; her symptoms did not improve and continued to worsen despite treatment. On directed questioning, she reported heavy menstrual bleeding, with cycles recurring every 21 days and menstruation lasting eight days. She had no known chronic illness and no history of caustic ingestion, radiotherapy, esophageal surgery, alcohol use, or smoking.

On physical examination, the patient appeared pale. There was no cervical lymphadenopathy, organomegaly, or palpable neck mass. Laboratory evaluation revealed severe microcytic hypochromic anemia, with a hemoglobin level of 7 g/dL (reference range: 12.0-16.0 g/dL), an MCV of 52 fL (reference range: 80-100 fL), and a ferritin level of 3 ng/mL (reference range: 15-150 ng/mL), consistent with profound depletion of iron stores.

The cause of the iron deficiency was investigated. The menstrual history described above was considered the most likely source of chronic blood loss. Fecal occult blood testing was negative. Colonoscopy was not performed, since a plausible source of blood loss had been identified and the patient had no lower gastrointestinal symptoms. The patient was referred to the gynecology department for management of menorrhagia; the treatment subsequently instituted there was not available to us.

Given the severity of the anemia, intravenous iron replacement was initiated. A total of 1,500 mg of ferric carboxymaltose was administered over two weeks, given as 500 mg in the first week followed by 1,000 mg in the second week. At the one-month follow-up visit, a marked hematological improvement was documented, with a hemoglobin level of 10.8 g/dL (reference range: 12.0-16.0 g/dL), an MCV of 71 fL (reference range: 80-100 fL), and a ferritin level of 776 ng/mL (reference range: 15-150 ng/mL).

To establish the etiology of the dysphagia, upper gastrointestinal endoscopy was performed. The arytenoids appeared normal. A web was identified at the esophageal inlet, producing marked eccentric narrowing of the lumen (Figure 1).

**Figure 1:**
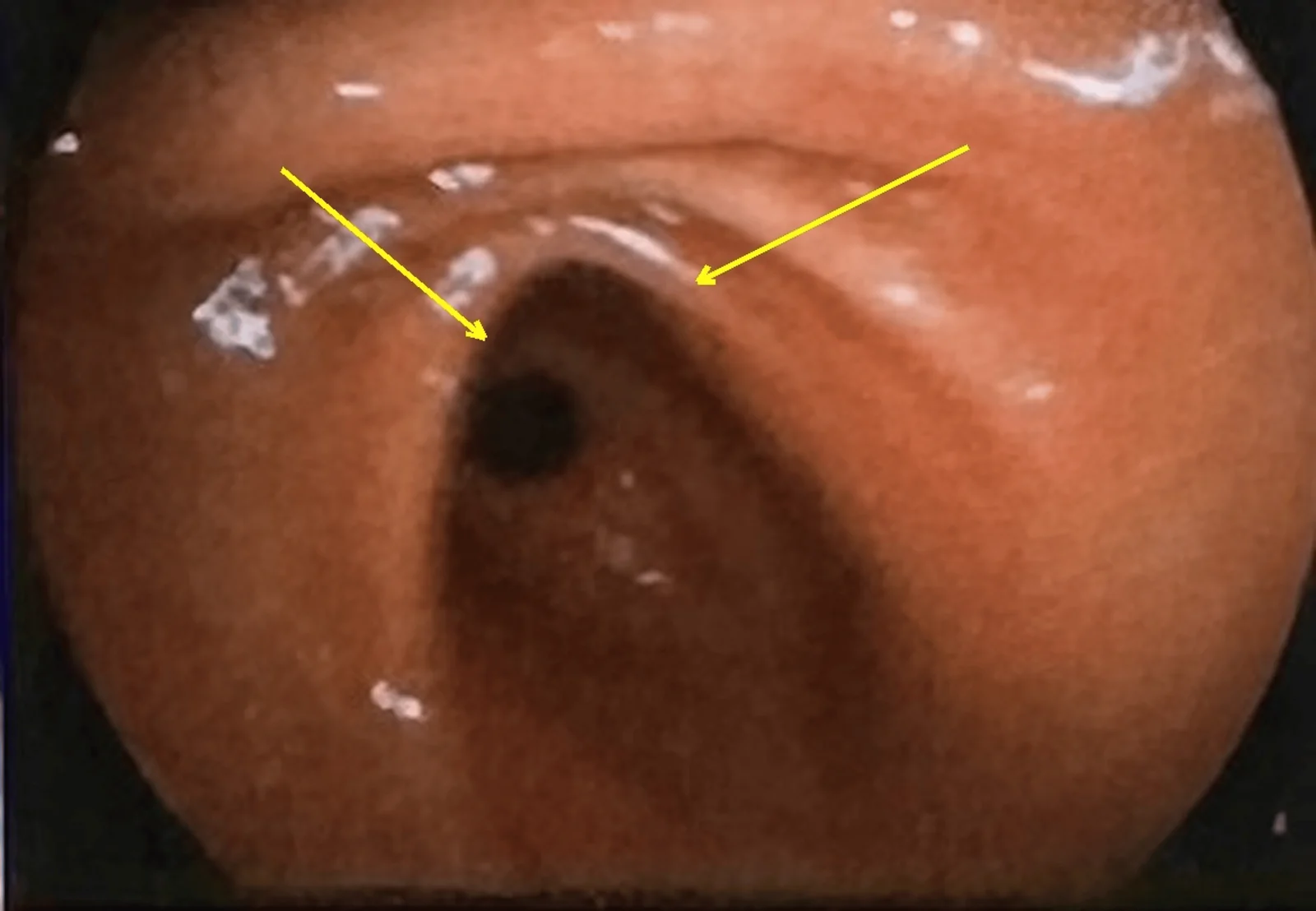
Upper gastrointestinal endoscopy prior to dilatation. The yellow arrows indicate the free margin of a thin, crescentic membranous web arising from the anterior wall at the esophageal inlet. The web extends across the lumen, reducing the residual passage to a small, eccentric opening between the arrowheads.

The endoscope could be advanced only with difficulty, and dilatation was performed during the same session using a combination of balloon and bougie dilatation. The final luminal diameter achieved was not recorded. No immediate adverse event, including bleeding or perforation, occurred. Following dilatation, the lumen was widened and radial mucosal fissures were visible at the site of the disrupted web (Figure 2).

**Figure 2:**
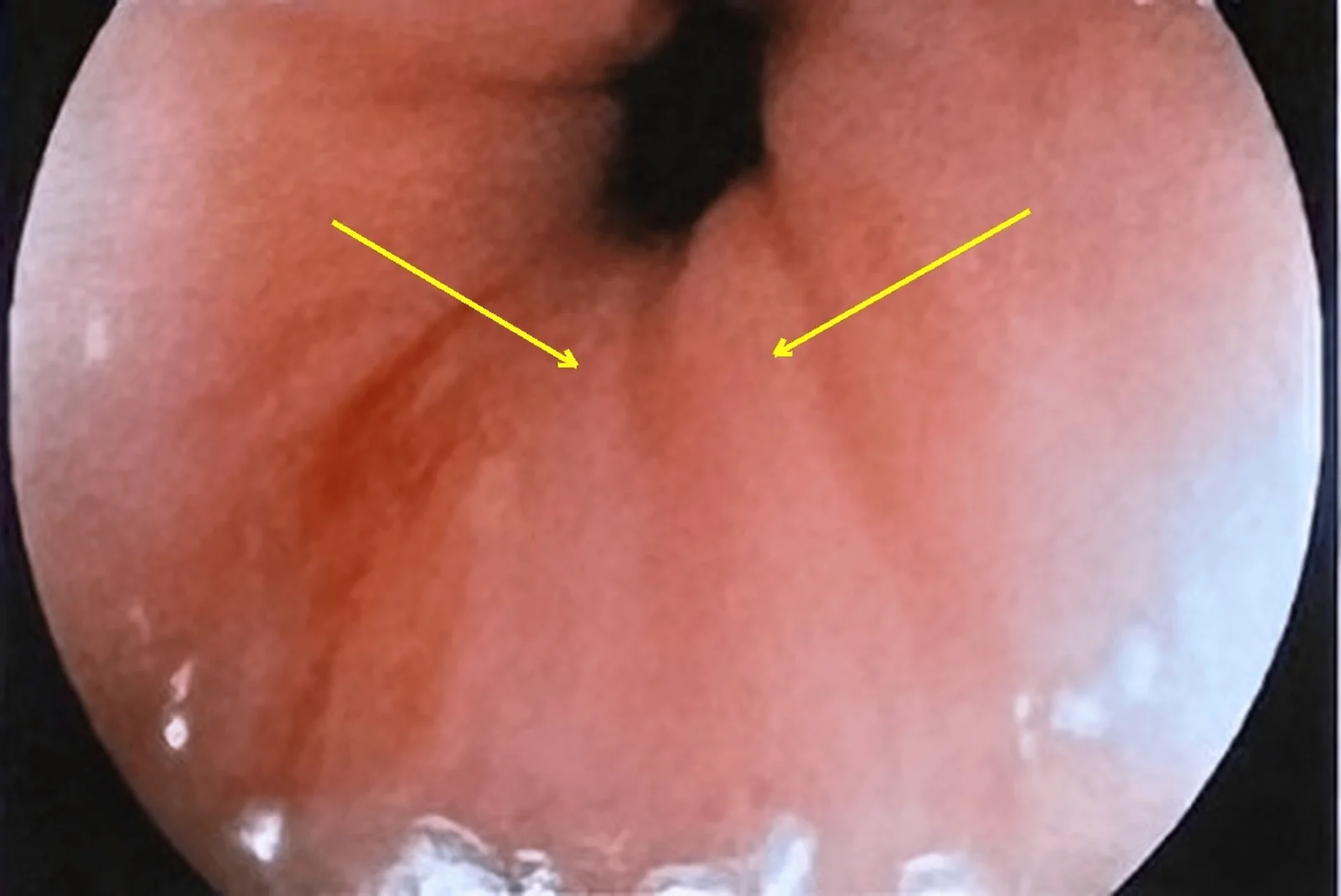
Endoscopic appearance immediately following dilatation. The yellow arrows indicate two linear mucosal fissures radiating from the widened lumen, corresponding to the sites of web disruption. The lumen is now patent, with no residual membrane visible.

The gastric mucosa, pylorus, and lower esophageal sphincter were normal, and no additional pathology was identified.

At the third month of treatment, a complete hematological response had been achieved, with a hemoglobin level of 13 g/dL (reference range: 12.0-16.0 g/dL), an MCV of 82 fL (reference range: 80-100 fL), and a ferritin level of 112 ng/mL (reference range: 15-150 ng/mL). The patient reported complete resolution of her dysphagia. She was enrolled in a long-term endoscopic surveillance program in view of the recognized malignant potential of the condition.

## Discussion

The present case reproduces the classical triad of PVS in a middle-aged woman, which corresponds to the demographic group most frequently affected [1,3]. The most instructive feature of the case, however, is not the diagnosis itself but the interval that preceded it. The patient received one month of empirical antisecretory therapy before either her anemia or her esophagus was evaluated. Empirical PPI treatment is a reasonable initial strategy for many upper gastrointestinal complaints, but dysphagia that is progressive or unresponsive to such therapy constitutes an alarm symptom and warrants prompt endoscopic assessment rather than continued empirical management [8].

The degree of iron deficiency observed here is also noteworthy. The combination of a hemoglobin level of 7 g/dL, an MCV of 52 fL, and a ferritin level of 3 ng/mL indicates long-standing and severe depletion of iron stores, which is congruent with the chronic mucosal changes described in PVS [2]. This observation carries a practical implication: a complete blood count obtained at first presentation is inexpensive, universally available, and may provide the decisive clue that prompts earlier referral in a patient whose dysphagia might otherwise be attributed to reflux.

Management of PVS rests on two complementary components. Iron replacement addresses the underlying deficiency and may alone relieve dysphagia in patients with thin, incompletely obstructing webs, whereas mechanical dilatation is required when the web causes significant luminal narrowing or when symptoms persist after repletion [1,7]. Endoscopic dilatation has been reported to be effective and safe in this setting, with symptomatic relief achieved in the large majority of patients [7,9]. In our patient, both modalities were employed, and the combination produced complete hematologic and clinical recovery. The choice of intravenous ferric carboxymaltose allowed rapid repletion of iron stores, an approach that is appropriate in patients with profound deficiency in whom oral therapy would be slow or poorly tolerated [10]. The markedly elevated ferritin recorded one month after parenteral iron should be interpreted with caution, since a ferritin level measured shortly after intravenous administration reflects the dose given and the acute-phase behavior of ferritin rather than stable iron stores; the third-month value is the more informative indicator of repletion in this patient.

Finally, the premalignant character of PVS merits emphasis. An increased incidence of squamous cell carcinoma of the hypopharynx and upper esophagus has consistently been reported in these patients, and surveillance endoscopy is therefore recommended [1,6]. Symptomatic recovery should not be interpreted as the conclusion of the clinical course, and patients should be counseled accordingly and enrolled in structured follow-up. A multidisciplinary approach involving internal medicine, gastroenterology, and otorhinolaryngology has been advocated to optimize both the initial management and the longer-term surveillance of these patients [6].

This report has several limitations. Observations derived from a single patient cannot be generalized, and the relative contributions of iron replacement and endoscopic dilatation to the resolution of dysphagia cannot be separated, since both interventions were applied within a short interval. Although menorrhagia and a negative fecal occult blood test made an occult gastrointestinal source unlikely, colonoscopy was not performed, and a coexisting lower gastrointestinal lesion therefore cannot be formally excluded. Finally, the gynecological treatment given for menorrhagia was outside our department and could not be documented, so the durability of the hematological response beyond the third month remains uncertain.

## Conclusions

Iron deficiency anemia should be actively sought in patients presenting with dysphagia that fails to respond to antisecretory therapy, and PVS should be retained in the differential diagnosis. A complete blood count obtained at first presentation provides an important early clue that prompts further investigation, although the diagnosis ultimately rests on endoscopic demonstration of the web. In the present case, the combination of intravenous iron replacement and endoscopic dilatation produced both hematologic and clinical remission within three months, and identification of the underlying source of blood loss allowed the cause to be addressed. Given the reported association with upper aerodigestive tract malignancy, long-term clinical follow-up with consideration of endoscopic surveillance is warranted even after complete symptomatic recovery, while recognizing that optimal surveillance intervals are not well established.
